# Barriers and facilitators for optimizing oral anticoagulant management: Perspectives of patients, caregivers, and providers

**DOI:** 10.1371/journal.pone.0257798

**Published:** 2021-09-29

**Authors:** Anne Holbrook, Mei Wang, Marilyn Swinton, Sue Troyan, Joanne M. W. Ho, Deborah M. Siegal

**Affiliations:** 1 Division of Clinical Pharmacology and Toxicology, Department of Medicine, McMaster University, Hamilton, Ontario, Canada; 2 Clinical Pharmacology Research, St. Joseph’s Healthcare Hamilton, Hamilton, Ontario, Canada; 3 Department of Health Evidence and Impact, McMaster University, Ontario, Canada; 4 School of Nursing, McMaster University, Hamilton, Ontario, Canada; 5 Division of Geriatric Medicine, Department of Medicine, McMaster University, Hamilton, Ontario, Canada; 6 Schlegel Research Institute for Aging, Waterloo, Ontario, Canada; 7 Division of Hematology, Department of Medicine, University of Ottawa, Ottawa, Ontario, Canada; University of Tennessee Health Science Center, UNITED STATES

## Abstract

**Background:**

Oral anticoagulants (OACs) are very commonly prescribed for prevention of serious vascular events, but are also associated with serious medication-related bleeding. Mitigation of harm is believed to require high-quality OAC management. This study aimed to identify barriers and facilitators for optimal OAC management from the perspective of patients, caregivers and healthcare providers.

**Methods:**

Using a qualitative descriptive study design, we conducted five focus groups, three with patients and caregivers and two with health care providers, in two health regions in Southwestern Ontario. An expert facilitator led the discussions using a semi-structured interview guide. Each session was digitally recorded, transcribed verbatim and anonymized. Transcripts were analyzed in duplicate using conventional content analysis.

**Results:**

Forty-two (19 patients, 7 caregivers, and 16 providers including physicians, nurses and pharmacists) participated. More than half of the patients received OAC for the treatment of venous thromboembolism (57.9%) and the majority (94.7%) were on chronic therapy (defined as >3 years). Data analysis organized codes describing barriers and facilitators into 4 main themes—medication-related, patient-related, provider-related, and system-related. Barriers highlighted were problems with medication access due to cost, patient difficulties with adherence, knowledge and adjusting their lifestyles to OAC therapy, provider expertise, time for adequate communication amongst providers and their patients, and health care system inadequacies in supporting communications and monitoring. Facilitators identified generally addressed these barriers.

**Conclusions:**

Many barriers to optimal OAC management exist even in the era of DOACs, many of which are amenable to facilitators of improved care coordination, patient education, and adherence monitoring.

## Background

Anticoagulants are the leading cause of medication-related serious harm as measured by emergency department visits, hospitalizations and fatalities [1, 2]. Each adverse drug event requiring a hospital visit approximately doubles the cost of care in the subsequent 6 months [3]. More than 30 million prescriptions per year are dispensed in North America for oral anticoagulants (OACs), which include warfarin, dabigatran, rivaroxaban, apixaban and edoxaban [4–6]. The high prevalence of OAC use among older adults, combined with their important clinical benefits in terms of reducing rates of stroke, embolism, and death, and their potential for causing major harm (primarily bleeds, which can be fatal), make them a medication safety priority.

Until the last decade, warfarin was the dominant OAC, with its own detailed requirements for management [7]. Multiple studies, with very few randomized trials, have addressed barriers and facilitators for high quality warfarin management [8]. Attitudes towards regular blood testing, concerns regarding adverse drug interactions, and the perceived requirement for very high INR time in therapeutic range, appear amongst the many proposed barriers and facilitators but are specific to warfarin [8]. As DOAC (direct acting oral anticoagulant–apixaban, dabigatran, edoxaban and rivaroxaban) utilization has markedly increased to become the dominant OAC in many countries, it is unclear if there are still unmet barriers in provider monitoring, patient knowledge, individual benefit:harm assessment, adherence, etc. [9–15] We set out to explore barriers to and facilitators for optimal OAC management from the perspectives of patients, caregivers, and healthcare providers. We defined optimal OAC management as that which leads to the lowest rate of adverse OAC-related events.

## Methods

### Design

We chose a qualitative descriptive study design with focus groups because it facilitates a summary of the data in the language used by participants with minimal theoretical interpretation–an approach recommended when straightforward descriptions of experiences are useful [16].

The protocol for this study was approved by Hamilton Integrated Research Ethics Board, and the Tri-Hospital Research Ethics Board for Kitchener-Waterloo-Cambridge. We followed the reporting recommendations of the consolidated criteria for qualitative research (COREQ) [17].

### Sampling and recruitment

We purposefully sampled patients and caregivers to include variation in their experience with OAC therapy–those currently taking OACs, those who had taken OACs in the past but discontinued taking them and also patients who had refused OAC therapy [18]. We attempted to recruit with balance of these factors, along with a range in age as well as the type of follow-up (organized OAC clinic, specialist clinic, primary care).

Similarly, we purposefully sampled a multidisciplinary mix of primary and secondary healthcare providers who prescribe, dispense or manage OAC therapy in community-based practices or hospital-based clinics in each of two cities. We planned for two focus groups each with patients/caregivers and healthcare providers (physicians of different specialties, nurses, pharmacists) ranging in size from 6–8 participants. Provisions were made for additional focus groups if saturation had not been reached [19].

Patients and caregivers of patients meeting any of the above-noted criteria were identified from practice lists of co-investigators in the two regions and were approached by telephone or email to see if they would be interested in participating. Healthcare providers were recruited via email or phone invitations by the investigators, with snowball sampling used until adequate numbers were recruited. Written informed consent was obtained from each participant at the beginning of the focus group discussion.

### Procedures

The focus groups were conducted in 2 cities (Hamilton and Kitchener-Waterloo) in Ontario, Canada. Each city represents a large health region with separate referral patterns, Hamilton including tertiary care hospital and clinic care [20]. The focus groups were held in the evening at convenient local community locations. Participants completed a brief demographic questionnaire after signing informed consent prior to the focus group discussion. The discussions were facilitated by an experienced focus group facilitator (MS) who has post-graduate training in qualitative methods, no conflicts of interest related to the topic and without any prior relationship to participants. She used a semi-structured discussion guide designed in advance with the other investigators (details in S1 Appendix). This approach provided some structure and guidance on the topics to discuss while also allowing for exploration of issues raised by participants that were not identified in the guide. Two research assistant non-participants were present to assist with logistics and to create some field notes, particularly related to participant ‘body language’ that was not in the direct line of sight of the facilitator. Each focus group lasted approximately 2 hours and was audio recorded. No follow-up or repeat interviews were undertaken.

### Data analysis

Demographic data were summarized using descriptive statistics. Data collection and data analysis occurred simultaneously to allow for the inclusion of new themes in the early data to be incorporated into the collection of later data. We used field notes from the focus groups to identify new themes to explore in future focus groups and we began coding after each focus group. We also conducted a preliminary analysis of the transcripts from the first two focus groups (one with patients/caregivers, one with providers) and added new ideas and insights as probes into our focus groups guide for the remaining focus groups.

The transcripts from the focus groups were analyzed using conventional content analysis, an analytic method that is based on an inductive approach to coding, with codes developed directly from the data rather than through the use of preconceived categories [21]. The analyst (MS) completed line by line coding of one transcript from each type of focus group (patient/caregiver and healthcare provider) highlighting words in the text that captured key thoughts or concepts and then developed code labels for those thoughts/concepts. These codes were organized into a preliminary list which was reviewed with other members of the research team who had reviewed but not coded the same two transcripts. This process was reproduced by another analyst to guarantee the rigor. Through discussion, a list of codes was developed which was applied to the remaining three transcripts. When coding was complete, the research team reviewed coding reports and met to organize the codes into meaningful categories based on their relationships to each other. The analyst ensured that there was at least one exemplar from the data for each code and category and recorded all decisions related to the coding and analysis process in a study audit trail [22]. Categories were collated into themes that we had previously developed in our scoping review [8]. The research team assessed data saturation through a review of all transcripts, coding reports and by examining the audit trail. NVivo (v11.0 QSR International, Australia) was used to manage the qualitative data for analysis.

## Results

### Participants

The study included 3 focus groups of patients and caregivers (N = 19 patients and 7 caregivers, mean of 9 participants per group). The mean age of these focus group participants was 62.2±13.9 years, ranging from 30 to 77 years old, and 14 were female (Table 1). The majority of the participants were currently using OACs (18/19, 94.7%), many for more than 3 years. The most common reason for using the OAC was previous venous thromboembolism (11/19, 57.9%) and most patients had their OAC therapy monitored by regular clinical visits.

**Table 1:** Participant characteristics.

Item	Number (%)
Participants	42
Patients	19 (45.2)
Caregivers	7 (16.7)
Physicians	9 (21.4)
Pharmacists	4 (9,5)
Nurses	3 (7.1)
Age (yr), mean (SD)	
Patient/Caregiver	62.2 (13.9)
Provider	48.4 (8.6)
Sex (female)	
Patients	14 (73.7)
Providers	12 (66.7)
Patient OAC Use Type (for 19 patients)	
Previous user	1 (5.3)
Current user	18 (94.7)
Duration of OAC Use	
0–6 months	8 (42.1)
More than 6 months to 1 year	0 (0)
More than 1 year to 3 years	2 (10.5)
More than 3 years	9 (47.4)
Indication for Use	
Atrial fibrillation	5 (26.3)
Previous venous thromboembolism	11 (57.9)
Mechanical heart valve	3 (15.8)
Number of previous thromboembolic events (venous or arterial), mean (SD)	1.05 (1.3)
Number of previous bleeding events, mean (SD)	0.11 (0.5)
OAC Monitored by*	
Family Physician	4 (21.1)
Specialist (e.g., hematologist, cardiologist or internist)	17 (89.5)
Provider Type of Practice (for 16 providers)	
Inpatient	1 (6.3)
Outpatient	7 (43.7)
Both	8 (50.0)
Provider Practice Location	
Urban	15 (73.7)
Rural	1 (6.3)
Provider OAC Management Activities (> 1 choice allowed)	
Prescribe OACs	13 (68.4)
Dispense OACs	4 (21.1)
Supervise OAC management	18 (94.7)
Advise other providers on OAC management	8 (42.1)

*Two of the patients were monitored by both Family Physician and Specialist.

Of 41 health care providers who initially agreed to participate (17 in Hamilton area and 24 in Kitchener-Waterloo area), 16 attended the focus group discussion with the rest citing schedule conflicts. The 2 focus groups included an average of eight participants (range 6–10) with a mean age of participants of 48.4± 8.6 years, and 12 females (Table 1). Providers included 4 pharmacists (25%), 3 nurses (19%), and 9 physicians (56%), with a range of involvement in OAC management including prescribing OACs (13/16, 81.2%) and supervising OAC management for their patients (12/16, 75%).

### Theme development

Codes developed during conventional content analysis were organized into four main themes according to their impact on OAC management: medication-related factors, patient-related factors, provider-related factors and system-related factors [8]. These are summarized in Table 2.

**Table 2:** Results themes and subthemes.

Theme	Barrier/Facilitator	Subtheme
Facilitators	Barriers	Doses per day
		Cost of OACs and insurance coverage
		Cost of monitoring
		Inconvenience of regular INR monitoring
		Difficulties interpreting laboratory results
		Lack of availability of reversal agents for DOACs
	Facilitators	DOAC instead of Warfarin
		Warfarin advantages
Patient-Related Factors	Barriers	Lifestyle
		Impact on financial-related benefits
		Cognitive impairment
		Mental health issues
		Lack of knowledge about OACs
		Addictions
	Facilitators	Belief in Effectiveness of OACs
		Patient Education
		Safety alerts
		Reminders
		Easy Access to OAC Provider Expertise
Provider-Related Factors	Barriers	Lack of Knowledge or Evidence
		Lack of Appreciation for Demands of Good Patient Follow-up
		Problems with Provider—Provider Communication
		Time Constraints
		Conflicting Recommendations from Providers
	Facilitators	Use of Decision Support.
		Accurate, Well-informed Discussion at Time of OAC initiation
		Encourage Open, Ongoing communication Between Visits
		Structure Follow-up Visits
		Reminders
		Use Strategies to Improve Compliance
		Develop a Relationship with the Pharmacist
System-Level Factors	Barriers	The System does not Support Good Communication
		Lack of Case Management Support
		Barriers to Timely Patient Information
		Work Schedules Not Supporting Continuity in Care
		Medication Shortages
		Discordant Appointment Expectations
	Facilitators	Shared Hospital Electronic Heath Record
		Embed Specialists in Primary Care Organizations
		Anticoagulation Clinic Services
		Flexible Laboratory Services
		Clear System for Communication

#### 1. Medication-related factors

*Medication-related barriers*. a) Doses per day. Both patients and providers noted that OACs that require more than one dose a day might pose a barrier. One provider hypothesized that "*I think that when some of the newer agents are dosed twice a day*, *you would probably see a decrease in compliance*.*”* One of the patients shared challenges she experienced related to an OAC that was dosed twice a day: "*Apixaban is two (doses per day) and I think I have missed my doses if I get busy in the morning… I have an alarm in my phone but then I press ’snooze’ and I forget*.*"* Even though warfarin is dosed daily, participants noted confusion related to dosing regimens requiring two different tablets “*I had a patient who had 5 milligram and 1 milligram tablets and rather than have two vials*, *they just put them together to just have one*. *So*, *her INR would be all over*.”

b) Cost of OACs and insurance coverage. Both patients and providers reported that insurance coverage for drugs could be a barrier to the optimal prescription of OACs. One patient described how coverage influenced the type of OAC that was prescribed *"A discussion that I’ve had with hematologists in the past has been*, *"Well*, *do you have coverage*?*"… "Oh*, *you have coverage*. *Okay*, *we’ll put you on Drug X" because it’s more expensive…maybe I don’t have coverage at this time*, *okay*, *well warfarin”*. A provider explained that "*for patient with venous thrombosis*, *we have a bit of an issue with DOAC coverage… There are patients -they tend to be younger … that becomes an issue and then even people who are over sixty-five*, *if the decision is for them to be on it longer*, *sometimes coverage determines how we look after them*.*”*

c) Cost of monitoring. The cost associated with follow-up appointments was mentioned. A patient noted "*It’s a big deal to go down there [to hospital]*, *you have to pay for parking …it’s half a day off work*.*…*" A provider also noted: *"We’re lucky enough to have point of care at the clinic*, *which is lovely*, *so they can just come in and it’s same day results*. *But we’ve moved buildings and now we charge for parking …so now we’ve got patients going back to the labs because it’s easier for them*. *So*, *the actual*, *just physically*, *getting an INR done can be difficult*.*”* For house-bound patients, the cost of having INRs done in the home was also identified as a barrier, *“It’s thirty dollars a time to have the labs come into their homes*.*”*

d) Inconvenience of regular INR monitoring. Several patients discussed this. One patient described: “*I was very*, *very unstable with my INR -to get out every week and…and have my blood tested was… I didn’t want to do it*. *So*, *there was actually a point in my therapy on blood thinners where I think I went six months that I didn’t go to get my INR tested*. *I was promptly switched to Xarelto after that*.*”* Another patient explained why he switched to a DOAC: “*I had to get periodic lab blood tests (INRs) and that was a big hassle because I would have had to get my wife to taxi me to the lab—I thought that would be too onerous for her*, *given all the other caretaking she had to do*, *so yeah*, *that was the consideration*.*”*

e) Difficulties interpreting laboratory results. A patient shared their experience: *I had a clot while on warfarin and they said it was like a false reading (INR) that sometimes you can have when you’re first getting therapeutic on it*. *It goes up—shoots up then goes back down … even a risk while we’re on it*. *Yeah*, *there can be negative aspects to blood thinners*.”

f) Lack of availability of reversal agents for DOACs. A few patients noted this issue, for example: “*So*, *your Rivaroxaban and your Apixaban or your Eliquis and your Xarelto…if I fall down the stairs*, *you know*, *there’s no way to reverse that effect…*” and “*I’m comfortable with Warfarin*, *it’s working*. *I know that if*, *somehow*, *I screw up on the dosage there’s an antidote*. *Some of the products that are out there on the market today*, *there is no antidote*.”

Providers shared their frustrations about other medication-related barriers including: the inability to perform therapeutic drug monitoring with DOACs, challenges related to co-morbidities and concomitant medications, the loss of trust from a patient after a drug interaction occurs and, a number of challenges related to changing patients from warfarin to DOACs.

*Medication-related facilitators*. a) DOAC instead of Warfarin. Compared to Warfarin, DOACs were considered as a facilitator for OAC management as they are easy to go on and off for short washout time and are not affected by diet. Several patients mentioned this benefit, for example: “*So*, *I find the Xarelto very good*. *There’s no testing or anything and it’s fairly easy to go on and come off it*.”

b) Warfarin advantages. Warfarin was noted to have the advantages of daily dosing, a medication compliance check in the INR test, a convenient point-of-care INR test, reversibility of anticoagulant effect, and low cost. Onepatient described: “*It’s the cheapest thing*. *So*, *that’s why I originally started with it*. *And that’s my choice of all my drugs*.*”* A physician described how when patients experience the bleeding with a DOAC, they may even lose their confidence to their physicians: “*…as long as you take your medication with the warfarin*, *you know*, *there are other checks that are happening so usually everything was hunky-dory*. *Not always*, *but most of the time*. *And then when they make the switch over to this newer medication and then there’s an adverse event that happens*.*”*

#### 2. Patient-related factors

*Patient-related barriers*. a) Lifestyle. Patients complained about how their lifestyle affects their adherence, for example, “..*if you’re on a medication where you have to take it at work*, *say you’re supposed to take it at eight o’clock every day…you get busy doing something*, *you forget to take it*, *you take it later in that day*. *So*, *lifestyle is definitely a barrier to the medication*, *to taking it on time or taking it consistently or the way it is prescribed*.” Another patient shared, “*Where I mess up is if something interrupts my normal schedule*, *something happens in the family*, *and then all of a sudden*, *I race off or something*, *I’ll miss a day*.*”*

b) Impact on financial-related benefits. One patient’s caregiver shared how they were declined mortgage insurance and told, “*Once you go off warfarin for one year*, *then we can insure you*.” She noted, “*they didn’t seem to get the lifesaving aspects of being on a blood thinner*.” Also, short-term disability was denied because of a blood clot even while on treatment, “*because they called it a [pre-existing condition] then there was a chunk of days they didn’t pay for*.”

c) Cognitive impairment. Providers identified this as a major contributor to compliance problems. A primary care physician described *“[We] pick up on people [who] once were really compliant and if they are not*, *it starts…signaling cognitive issues*.” Another provider noted “*There are older patients that are still on their own and might not have family support yet but they’re on the cusp of nursing home [and] starting to diminish*.” A geriatrician noted the important role of caregivers to monitor the patient and their adherence.

d) Mental health issues. One provider described how younger patients who have had mechanical valves implanted but also have mental health issues “*just disappear off our radars*”, while another provider explained that “*anxiety*, *depression*, *can really influence people in terms of stopping medication or their anxiety of taking medication*.”

e) Lack of knowledge about OACs. One provider explained: *“*…*often non-compliance is about lack of understanding of risk and what the drug is doing and not doing to protect them*.*”*. Another provider described how when the OAC is being used to prevent a stroke in a patient who has atrial fibrillation but has not had a stroke, the patient “*may not really understand what the implications of that actually are*. *It can be very difficult*, *I think*, *for patients to understand abstract concepts around there’s a blood clot that forms in your heart and can fly off into your brain*. *I think people have a hard time with that*. *So*, *sometimes that can affect people’s motivation*.*”* Providers also worried about the lack of retention of OAC-related education: “*I’ll see in a note that’s dictated that we definitely discussed the risks and benefits of this type of medication for a stroke prevention*, *and the patient will say*, *‘I don’ remember that at all*,*” or*, *“Yes*, *I remember them kind of talking about it but I wasn’t really able to take in that much just because of everything that was happening*.”

f) Addictions. Caregivers and providers both mentioned alcohol as interfering with medication compliance and INR time in therapeutic range. A provider gave the example of *“…a binge drinker*, *their INRs will go sky-high on us which means their blood is really thin… then they go into a nursing home and all of a sudden their INR falls really low and we can’t figure out why*. *And it’s because they’re not drinking anymore*.*”*

*Patient-related facilitators*. a) Belief in Effectiveness of OACs. The patient’s belief that OACs can improve and maintain their life was a frequently mentioned facilitator. Patients described how OACs allowed them to live and get back to a better quality of life with decreased risk of potentially fatal outcomes such as pulmonary embolism (PE) or deep vein thrombosis (DVT). As one patient stated, “*I guess the benefits are*, *you know*, *live to see another day*.*”* A few patients also described how taking an OAC gave them peace of mind and decreased their anxiety that every ache could be a blood clot. One patient explained “*one of the benefits of being on the blood thinner medication was that sort of peace of mind*, *or that sort of decreased anxiety*.*”* Another facilitator identified by patients was convenience, with patients describing how some DOACs are more convenient than Warfarin and how OACs are more convenient than heparin-type injections.

b) Patient Education. Several patients reported that educating themselves about their condition and their OAC (e.g., how the medication works, and its benefits, harms and dosing) was important for management. Patients realized the importance of OAC knowledge for them: “*Patient knowledge is one of the biggest things because if you don’t know*, *you can’t advocate for yourself*.*”* Providers agreed with this point of view: “*I think the most important factor there would be people who truly understand the outcomes when they don’t take medication properly*. *That they actually understand how the medication is working and what it’s really doing*.*”*

c) Safety alerts. One patient reported that always carrying the information that they are taking an OAC with them could be lifesaving if they are unable to speak. One patient reflected, *“I don’t know if there’s anything other than carrying the information in my pocket all the time that I’m on blood thinners*.*”*

d) Reminders. Patients and providers noted that reminders of different kinds were very helpful for them, including carrying an emergency dose in a key latch, keeping medication in visible locations, keeping pills in a purse or bag, using pill organizers, and using dose reminder alarms (cell phone or pill organizer with built in alarm). For instance, a patient described her pill organization “*I usually set a day pillbox*, *three sections per day*, *and my warfarin goes in the bottom one because I always take it in the evenings*.*”*

e) Easy Access to OAC Provider Expertise. This included access to laboratory resources, and providers who could advise on: dosing changes, stopping and re-starting the OAC for dental work, and drug and food interactions. A patient reported: “*Having the medications and the kind of monitoring we have available…*.*I think it’s a real blessing and a real privilege*.*”* Several providers noted the helpfulness of an anticoagulation service, for instance: “*I also think one of the things that is helpful is support from the managing clinic*. *So*, *follow-up phone calls*, *I find that kind of thing helps*”

#### 3. Provider-related factors

*Provider-related barriers*. a) Lack of Knowledge or Evidence. Providers acknowledged that they did not always feel confident about how to proceed with anticoagulation management. For example, one physician shared”*I see a lot of patients who are…getting unwell almost to the palliative point and having those conversations about*, *you know*, *risk/benefit*, *continuing anticoagulation at some stage of their life…I think it’s one of the last things to get stopped*.”

b) Lack of Appreciation for Demands of Good Patient Follow-up. Providers highlighted OAC therapy required ongoing follow-up due to changes in the patient’s health status, stage of OAC therapy, concomitant medications, medications available, or changes in the research evidence. For example, “*I think [patients] need to be reminded that anticoagulation discussion is an ongoing discussion and that as more evidence mounts*, *our recommendations may change—may very well change for their individual case*.

d) Problems with Provider—Provider Communication. Both family medicine physicians and hospital based specialists described challenges communicating with providers outside of there are of practice. A family physician noted “*I don’t know if specialists are as afraid to call us*, *timewise*, *as we are to try to get in touch with them*. *We don’t want to take time away from them*, *and them from us and that sort of thing*.” Similarly, a specialist shared “*I’ve definitely had to kind of try to dodge the receptionist a few times and been*, *like (sternly)*, *“I am Dr*. *So-and-So calling from this hospital for this patient that has been admitted*… *and I have no other information…I think access [to clinical information] is important*.

e) Time Constraints. Providers described challenges with time available for appointments, for example “*The patient shows up with a list of fifteen things and is seeing the pharmacist for the INR [only] but because they are in the physical space of the clinic they want*, *you know*, *fourteen other things checked as well*, *but they’re not booked and there’s nobody to address that*.”. Another physician noted *“…a lot of times you’re paying attention to recurrent symptoms or bleeding or what-not*. *Not often do we spend a lot of time talking about*, *“Are you taking your medicines*?” Physicians also noted the additional work that OAC management required outside of appointments “..it’s the family doc calling them [patients] for the INR results…that seems to me to be an onerous amount of work.” Competing demands were always in play: “*If somebody’s INR is way different than it was last time*, *to try to unravel that at six o’clock at night is challenging”*

f) Conflicting Recommendations from Providers. A patient from Hamilton complained about receiving controversial recommendations from different physicians: “*The neurosurgeons at were saying to me*, *“Oh yes*, *there’s absolutely no reason you need to take this Warfarin*. *You should go off of it*.*” And I said*, *“But my doctor says my D-dimer is so high*.*” And they were like*, *“Oh no*, *that’s ridiculous*.”

*Provider-related facilitators*. a) Use of Decision Support. Providers reported “*something that I do behind the scenes is I use an algorithm-based dosing software that calculates individual time of therapeutic range”* and described how “*It can kind of flag you to say the trend is going in this direction; you need to be paying a bit more attention*.”

b) Undertake Accurate, Well-informed Discussion at Time of OAC initiation. For instance, a physician mentioned *“…I really spend a lot of time at the beginning helping the patient to understand the medication that they’re taking and the reason that they’re taking it*”.

c) Encourage Open, Ongoing communication Between Visits. As one of the nurses said: “*We do try to get our patients to call us so*, *for example*, *anybody changes your medications*, *you need to call us*.”

d) Structure Follow-up Visits. Providers described encouraging patients to bring all of their medications to each appointment, and recommending that patients keep a journal of changes and events, noting that, *“they can keep track of things because there often is a lot to keep track of*.*”*

e) Reminders. Providers believed that phone calls to patients were helpful to remind them to take medications or find out why follow-up appointments were missed.

f) Use Strategies to Improve Compliance. Providers described using different strategies to improve OAC compliance including: asking the patient about missed or extra doses, providing them with a calendar with written instructions for tracking medication use, ensuring the patient has refills on their prescription, and encouraging the use of dose organizers. One provider explained: “*we give calendars out to patients with doses written down versus verbal instructions*.” Another provider noted: “*We give handouts*, *now there’s Thrombosis Canada handouts*…*”*

g) Develop a Relationship with the Pharmacist. One physician described: *“In my practice*, *[I] actually make that call…That goes a long*, *long way to establishing that personal relationship with the community pharmacist who sees them much more than we do*.*”*

#### 4. System-level factors

*System-related barriers*. a) The System does not Support Good Communication. Patients, caregivers and providers reported expectations that their healthcare system should improve communication especially between doctors, between doctors and pharmacists, and in the patient’s transition from hospital to the community. One of the physicians said: “*It is really difficult to communicate from hospital [to community] …or even from doctor to doctor…in real time about what’s happening*.” Understanding current medications was seen as essential but family doctors expressed frustration with documentation they receive from the hospital, “*there’s still the nightmare of that discharge paper that comes with the five-page medication list that is so confusing*” and with lack of inclusion in important communication: “the *hospital team may fax the discharge prescription straight to the community pharmacy …but does that actually get back to the family doctor …*.*no*.*”*

b) Lack of Case Management Support. One of the providers noted: “*I think that we have to be realistic and know that case management approach is not available to a lot of patients*. *Solo family docs … are just not going to have that level of case management which*, *I think*, *will influence compliance*.”

c) Barriers to Timely Patient Information. Family physicians discussed challenges to accessing timely patient information: *“the [information]push would come to you but not necessarily in a timely manner whereas Clinical Connect [a regional hospital electronic health record] is real time but*, *of course*, *it’s time consuming*. *You have to go looking*.*”* Similarly specialists noted having insufficient information on patients to make good decisions, one reflected: “*we’ll be in bridging clinic and we’ll see a patient in there on an anticoagulant and I think*, *“I can’t really advise you when to restart this anticoagulant because I’m not sure why you’re on it in the first place*.*”*

d) Work Schedules Not Supporting Continuity in Care. A physician noted: “*So [as a patient] you’re seeing one person one day*, *another person another day*, *and you’re bringing them back in three days*, *and maybe they’re coming back in five days*, *and it’s different people making that decision all the time with different training and different agendas*.*”*

e) Medication Shortages. Both patients and physicians mentioned this issue. As one patient said: “*Sometimes they [the pharmacy] don’t have enough*, *don’t have any [of the prescribed medication]…So*, *that can impede taking the medication as prescribed by doctors*.*”*

f) Discordant Appointment Expectations. As one patient said: “*Please don’t tell me that I’m going to come down there and you’re going to [just] pat me on the back*. *Because it’s like*, *twenty bucks for parking and a half day of work lost*.” Similarly, providers shared: “*So*, *the patients that come to the INR clinic are often an issue because they also want other medical issues addressed at the same visit*.”

*System facilitators*. a) Shared Hospital Electronic Heath Record. Family physicians used the regional hospital system to follow patient admissions and flag patients being prepared for discharge, etc. For example, one of them stated: “*The other tool that facilitates is Clinical Connect*. *Because I know my patients that are admitted and I’m following their admission status*.”

b) Embed Specialists in Primary Care Organizations. A family physician noted: “*that model of embedding the specialists right into the outpatient primary care setting has been very successful for building the relationship with patients*. *Speaking to the challenge of the patient not wanting to take recommendations from anyone but the specialist……*, *it bridges that gap*.”

c) Anticoagulation Clinic Services. Patients and family physicians appreciated the expertise and support provided by anticoagulation clinics. Patients described: “*I am getting very good care at the Thrombosis Centre*…*really excellent care*”, and *“One of the things that is helpful is support from the managing clinic*. *So*, *follow-up phone calls*, *I find that kind of thing helps*. *“Another comment*, *“When people just want to ask a very quick question*, *they just pick up the phone and [the clinic] pharmacist is there to go*.”

d) Flexible Laboratory Services. Healthcare providers believed home and care home visits from laboratory services were helpful, as was point-of-care laboratory testing in the primary care office space.

e) Clear System for Communication. This includes communication from specialists to family physicians. One family doctor described, “*The best ones*, *for me…they (patients) have a little envelope for me*, *“This is exactly what I’m on*,*” and it says*, *you know*, *times two weeks*, *or whatever*, *so those ones are pretty crystal clear*.” Facilitators identified for a clear system for communication from the hospital to community included ensuring automatic notification of the patient’s discharge, facilitation of rapid dispersal of the discharge summary, and sending a copy of the discharge prescriptions. Participants expressed hope that a future innovation–ePrescribing, will improve pharmacist-prescriber communications, support adherence, and improve the quality of drug interaction alerts.

## Discussion

Our study sought wide-ranging, multi-disciplinary insights from patients, their caregivers, physicians (both primary care and specialists), nurses, and pharmacists on perceived barriers and facilitators to optimal oral anticoagulant management. We deliberately chose participants at 2 different types of health care delivery regions- a large, urban center with internationally acclaimed academic tertiary care expertise, and a smaller urban center with a community hospital and an active primary care network. Despite these different settings and patient care models, the concerns regarding OAC management were largely the same.

In this study, a large number of barriers were related to the OAC medication itself, to patient characteristics or limitations, to provider knowledge and style of practice, and to system-level of organization of care were identified with minimal disagreement between patient and provider groups. Few barriers were insurmountable and the facilitators suggested were mostly oriented to improved coordination of and communication around OAC management. Although DOACs created fewer barriers in some respects than warfarin (laboratory monitoring, for example), they were associated with new barriers as well (e.g., restricted public funding, lack of adherence check). The increasing number of new DOACs has increased the overall complexity of OAC management, due to different dosage regimens, indications, and pharmacologic characteristics.

A previously published scoping review of barriers and facilitators to optimal anticoagulation management by our group found 62 studies addressing this, including three randomized trials.(8) These studies predominantly address warfarin-related issues. In the review, we identified four theme categories–medication-related, patient-related, provider-related, health care system-related. Patient-related barriers were the most frequently mentioned and were more frequently shown to be associated with surrogate management outcomes, but no study addressed the most important outcomes which are clinical events including death, hemorrhages and thromboembolic events. Despite lack of patient education frequently being identified as a barrier, a recent systematic review of randomized trials examining the effect of supplemental patient education found low quality evidence of an improvement in knowledge scores but only a trivial improvement in clinical outcomes [23].

Our study has several strengths including practical design, recruitment of multiple stakeholders and achievement of data saturation. There are some limitations inherent in our design choices. Our study was conducted in the Hamilton and Kitchener-Waterloo regions, thus generalizability of our findings to other geographic areas, particularly rural and remote areas, may be limited due to differences in local practice and health system organization [24–26]. In addition, we were unable to recruit patients who had declined anticoagulant therapy. Finally, the recruited patients represented VTE disproportionately to atrial fibrillation.

This study has implications for practice, policy and research. Many of the barriers and facilitators are directly usable in clinical practice and could potentially improve patient knowledge and satisfaction with care. However, since there is no good evidence that interventions to improve barriers or implement facilitators to improve OAC management actually improve clinical outcomes, it would be premature to mandate policy towards these. Randomized trials to investigate whether interventions to improve coordination of care while incorporating other facilitators can improve patient-important outcomes, would be welcome.

## Conclusion

Barriers to optimal OAC management in the era of DOACs continue to highlight medication access and cost of drugs and monitoring, patient difficulties with adherence, knowledge and adjusting their lifestyles to OAC therapy, provider expertise and time for adequate communication amongst themselves and their patients, and health care system roles in supporting communications and monitoring. Facilitators addressing these barriers suggested improved care coordination, patient education, and adherence monitoring.

## Supporting information

S1 Appendix (DOCX)
