# Supplementary material for: Barriers and facilitators for optimizing oral anticoagulant management: Perspectives of patients, caregivers, and providers
Source: PLoS One. 2021 Sep 29;16(9):e0257798. doi: 10.1371/journal.pone.0257798 (PMC8480846; doi:10.1371/journal.pone.0257798)
Supplement: S1 Appendix — (DOCX) [file pone.0257798.s001.docx]

**Appendix. Focus Groups Topic Guides**

1. **Healthcare Providers.**

| **Domain** | **Topic** |
| --- | --- |
| **Introduction** | I’d like to begin by asking everyone to introduce themselves by name, and also describe their clinical specialty. |
| **Management of anti-coagulants** | **Health care provider’s perspective anticoagulant management**  1) We’d like to ask you begin by thinking about the patients you see who have the best adherence to taking the oral anticoagulants as prescribed.  Why are these patients doing well with taking their oral anticoagulants?  Probes:   - Patient-level factors (socio-economic status, ability to be compliant, willingness to be compliant, challenges accessing medical care including distance/transportation). - Support system - Think of one patient you provide care for who is doing well – what things contribute to their success with taking the oral anticoagulants?   2) What other behaviors do you believe are important for patient self-management of oral anticoagulants?  3) Based on your clinical experience, what aspects of healthcare provider management do you believe influence the management of oral anticoagulants?  Probes: patient follow-up  4) Summarize discussion about facilitators for adherence to oral anticoagulants.  Anything else to add about facilitators for adherence to oral anticoagulants?  5) Next, we’d like to ask you to think about which patients are not adhering to their oral anticoagulants. Why do you think these patients are not doing well with taking their oral anticoagulants?  Probes: patient-level factors (socio-economic status, ability to be compliant, willingness to be compliant, challenges accessing medical care including distance/transportation, risk of falling   - Need for more regular blood tests. - Adjusting/determining the correct dose, dietary restrictions. - Patient fear of risks associated with oral anticoagulants - Drug side effects - Drug interactions - Support system   Think of one patient that you provide care for who is having a difficult taking their anticoagulants as prescribed – what challenges do they deal with?  6) What are some of the difficulties that as clinicians, you experience with managing oral anticoagulant medications in your patients?  Probes:   - Adequate time and resources for patients to be informed? - Knowing if the patient is taking what they should be taking/prescribed to take? If yes, why is this difficult? - Challenges related to identifying individual patient risk factors for benefit versus harm? - Dose adjustments over time and around procedures – monitoring and reversal strategies - Issues around legal liability? - Issues around having different professions working together inter-professionally? (I.e., concern about other healthcare providers stepping on physician's toes/taking over their scope of practice; concern about the ability of other professions to manage patients taking oral anticoagulants?)   Think about one of the patients where you are had a very difficult time managing their oral anticoagulants – what made it difficult?  7) What would things help you to manage your patients' use of oral anticoagulant medications more successfully?  Probes:   - patient level factors - system level factors   8) Summarize discussion re: challenges to oral anticoagulant medication adherence.  Anything else to add to the challenges to oral anticoagulant medication adherence? |
| **Education** | **For the next part of the discussion, we would like to focus on patient education about oral anticoagulant medications.**  1) Can you begin by telling me how patients are educated about oral anticoagulants?  Probes:   - Discussions during visits for medical care, pamphlets - How do you educate the patients you provide care to about oral anticoagulant medications? - Do you believe patients receive enough education about 1. Their medical condition and the risk of stroke/blood clots; 2. The role of oral anticoagulant medications in lowering risk for stroke/blood clots; 3. The risks associated with taking oral anticoagulants (primary bleeds); 4. The importance of being compliant when taking oral anticoagulants - Do you think there is every any reluctance on the clinician’s part to educate patients about the risks associated with taking oral anticoagulants? - Is the education they receive relevant to them? (I.e., in the language they can understand and tailored to the patient experience?)   2) In your experience do you believe that most patients or their caregivers understand enough about oral anticoagulant medications?  Probes:   - why or why not   3) What are some of the challenges to educating patients about oral anticoagulants that need to be addressed?  4) Summarize discussion about OAC education.  Are there any other ways that patient education about oral anticoagulant medications can be improved? |
| **Commun-ication** | **For the last part of the discussion, we would like to focus on the role of communication in the management of oral anticoagulant medications.**  1) What types of communication are typically involved in the management of oral anticoagulants?  Probes:   - Face-to-face - Phone calls - Emails - Texts to and from patients - With other clinicians/healthcare providers - Communications from labs   2) Have you experienced any communication barriers related to the management of oral anticoagulants?  Probes:   - the communication breakdown with patients/caregivers - communication breakdown between clinicians involved in patient's care (i.e., one clinician prescribes a medication that will interact with oral anticoagulants b/c they aren't aware the patient is taking oral anticoagulant)   3) Have you experienced any factors that make it easier to management oral anticoagulants in patients?  4) How do you think communication-related to oral anticoagulant medications can be improved? |
| **Closing** | We are now approaching the end of our discussion. Is there anything else anyone would like to add to the management of oral anticoagulant medications?  Thank you for sharing your experiences, your perceptions and your time. |

**b) Patients and Caregivers**

| **Domain** | Topic |
| --- | --- |
| **Introduction** | I’d like to begin by asking each of you to introduce yourself by name, describe if you are a patient or a caregiver and share your experience with blood thinners (currently taking them, took them in the past, or refused to take them). |
| **Anticoagulant Knowledge** | For the first part of the discussion tonight, we’d like to talk about your knowledge about blood thinners.  1) Can you start by describing why you are taking blood thinners, or if you have taken them in the past or refused to take them, why your doctor suggested that you take them?  2) Next, can you describe some of the benefits of taking blood thinners?  3) And now can you please talk about some of the potential harms associated with taking blood thinners?  4) For those who have refused to take blood thinners, can you tell us why you refused to take them?  5) For those of you who are taking blood thinners or are a caregiver for someone who takes blood thinners, can you describe if you can take them the way the doctor has prescribed them for you?  If yes, why are you (or the person you provide care for) able to take blood thinners the way your doctor prescribed them?  Probes:   - What things make it easy to take blood thinners? - Motivation - Comfort level with blood thinners - Trust in doctor - Support from family/friends - Support from health care providers/clinic (including follow-up via phone or at visits) - Clinic/lab easy to get to - Reminders: calendars, alarms on phones, dockets, blister packs, et al. - Routine   Have you ever thought about stopping your blood thinner medication?  If yes, why have you thought about stopping your blood thinner medication?  If yes, why haven’t you stopped taking your blood thinner medication?  For those of you did stop taking blood thinners would you willing to share with us the reasons why you stopped taking them?  If no, why aren’t you (or the person you provide care for) able to take blood thinners the way your doctor prescribed them?  Probes   - What things make it difficult for you to take blood thinners? - Lack of motivation (including knowledge) - Discomfort with blood thinners (INR testing, potential risks including risk of falling) - Dose adjustments over time and around procedures – monitoring and reversal strategies - Side effects experienced from taking blood thinners - Blood thinners interact with other medications - Lack of trust in doctor - Lack of support from family/friends - Lack of support from health care providers/clinic (including no follow-up or limited follow-up via phone or at visits) - Challenges with getting to lab/clinic/doctor (distance, transportation, working hours, physical mobility challenges)   What do things you think would make it easier for you to take blood thinners as prescribed?  Probes:   - Communication from healthcare team (face-to-face, phone calls, emails, texts). Probe re: preferred method of communication; if/how communication might make it more difficult rather than easier?   Have you talked about any of the challenges you experience with taking blood thinners with your doctor or healthcare team? Why or why not?  Does your doctor know you aren’t taking your blood thinners as prescribed?  Why or why not? |
| **Education** | For the second part of the discussion, we would like to talk about education about the use of blood thinners.  1) Can you tell us about the education you received about/information about blood thinners?  Probes:   - What did you learn about? - Your medical condition and the risk of stroke/blood clots - The role of blood thinners in lowering risk for stroke/blood clots - The risks associated with taking blood thinners (primary bleeds) - The importance of taking blood thinners as prescribed (being compliant) - Who? - When? - How? (pamphlets, discussion, website/video) - How often? - Was the education presented in language that you could understand?   2) Do you feel you know enough about the blood thinner that you are taking or that your doctor recommended to you?  If no, what things would you like to know more about?  3) Do you have any suggestions about how we could improve the way patients and caregivers are educated about blood thinners?  Probes:   - Who? - When? - How? (pamphlets, discussion, website/video) - How often? - What kind of information is shared? - Language used? |
| **Commun-ication** | 1) What communication (face-to-face, phone calls, email) would you think helps to ensure the medication you are taking are managed in the best possible way?  2) What communication would make management more difficult?  3) Are there any suggestions you have that could improve this? |
| **Closing** | We are now approaching the end of our discussion. Is there anything else anyone would like to add to their experience with blood thinners that we have not talked about?   - Summarise - Thank participants - Provide extra information and contacts to participants |
